# Not a Lost Art: A Practical Guide to Key Techniques and Recent Innovations in Protist Culturing

**DOI:** 10.1111/jeu.70140

**Published:** 2026-11-13

**Authors:** Daniel J. Richter, Ivan Čepička, Javier del Campo, Stefan Geisen, Vladimír Hampl, Anna Karnkowska, Enrique Lara, Julius Lukeš, Kasia Piwosz, Ian Probert, Cecilia Rad‐Menéndez, Alastair G. B. Simpson

**Affiliations:** ^1^ Institut de Biologia Evolutiva (CSIC‐Universitat Pompeu Fabra) Barcelona Spain; ^2^ Department of Zoology, Faculty of Science Charles University Prague Czech Republic; ^3^ Laboratory of Nematology Wageningen University & Research Wageningen the Netherlands; ^4^ Department of Parasitology, Faculty of Science Charles University, BIOCEV Vestec Czech Republic; ^5^ Institute of Evolutionary Biology, Faculty of Biology University of Warsaw Warsaw Poland; ^6^ Department of Mycology Real Jardín Botánico‐CSIC Madrid Spain; ^7^ Institute of Parasitology, Biology Centre, Czech Academy of Sciences, and Faculty of Science University of South Bohemia České Budějovice Czech Republic; ^8^ Department of Fisheries Oceanography and Marine Ecology National Marine Fisheries Research Institute Gdynia Poland; ^9^ Roscoff Culture Collection, FR2424 Station Biologique de Roscoff Sorbonne University/CNRS Roscoff France; ^10^ Culture Collection of Algae and Protozoa Scottish Association for Marine Science Oban Scotland, UK; ^11^ Institute for Comparative Genomics and Department of Biology Dalhousie University Halifax Canada

**Keywords:** cultivation, culture media, isolation, long‐term maintenance, protist, trophic modes

## Abstract

Protists represent the majority of eukaryotic diversity and play essential roles in ecosystems worldwide. Obtaining protist species in culture permits in‐depth studies that provide insights into their cell biology, physiology, and biochemistry, and can transform them into valuable laboratory models representing lineages beyond plants, fungi, and animals. Despite a long history of protist cultivation, the vast majority of protists remain uncultured. This practical guide provides a thorough reference for current knowledge on protist cultivation. It includes information on approaches for initial isolation, selection of growth vessels and storage conditions, optimization of culture media, and adaptation of cultivation strategies to the known or hypothesized trophic modes of target species. We also discuss the importance of associated organisms, including prey, symbionts, and microbiomes, in successful cultivation. In addition, we summarize current approaches to long‐term maintenance and cryopreservation, which remain technically challenging but offer substantial practical benefits. Finally, we highlight emerging technologies and methodological innovations that could drive major advances in future protist cultivation efforts. We anticipate that a renewed focus on protist cultivation will accelerate the discovery and experimental study of diverse protist lineages, expanding their use as model systems and deepening our understanding of eukaryotic diversity and evolution.

## 1 Introduction

### 1.1 Why Should Protists Be Cultured?

Protists represent the majority of eukaryotic diversity (Figure 1) and inhabit a wide range of environments, including marine and freshwater systems, soils, extreme environments, and host‐associated niches. The immense diversity of protists underlies the roles they play in global ecosystem functions. Phototrophic protists are major players in the carbon cycle, both in aquatic and terrestrial systems (Worden et al. 2015; Jassey et al. 2022). Heterotrophic protists influence the cycling of carbon and other elements through predation on bacteria, fungi, and other microbial eukaryotes; this microbial food web has long been acknowledged and is of major importance in recycling nutrients (Azam et al. 1983; Clarholm 1985). In soils, nutrients liberated by protists are important for plant growth, making them key contributors to soil fertility (Geisen et al. 2018). Protists also play major ecological roles as symbionts, encompassing mutualistic, commensal, and parasitic relationships that strongly influence their hosts and partners. From a human perspective, these interactions are often viewed negatively because protist pathogens can have severe impacts on humans, livestock, and crops. However, parasitic interactions can also enhance ecosystem stability and complexity by preventing overdominance of particular host species (Berlinches De Gea et al. 2025). Mutualistic protists directly benefit their hosts, as illustrated by lichen‐associated microalgae or parabasalian symbionts of termites. In addition, some species of protists have important biotechnological applications and contribute to the development of new bioproducts (Berlinches De Gea et al. 2025). Collectively, protists are therefore fundamental to biosphere functioning.

**FIGURE 1:**
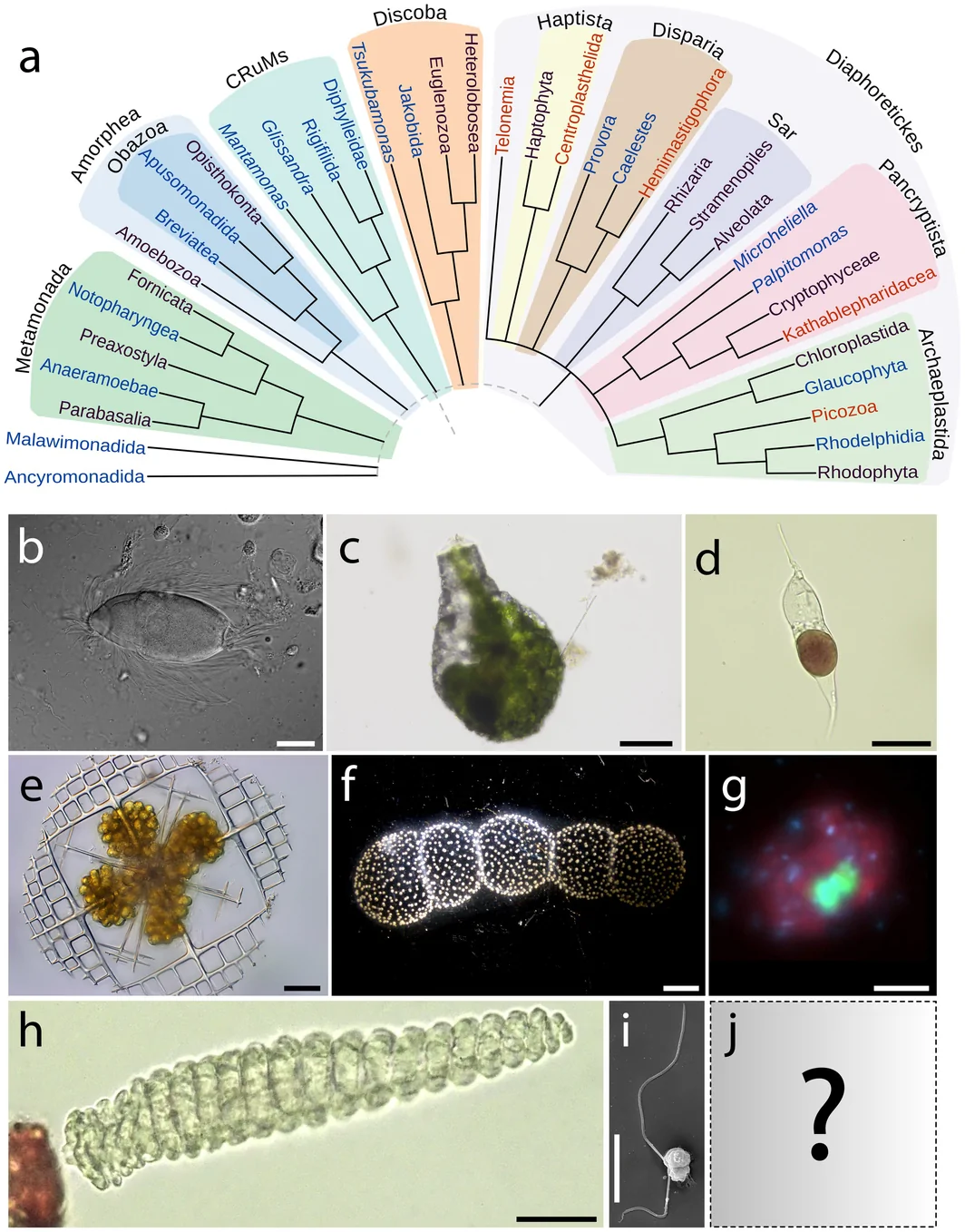
Known eukaryotic diversity based on recent publications (Schön et al. 2021; Williams et al. 2024; Torruella et al. 2025; Shiratori et al. 2026; Valt et al. 2026), and a subjective “most wanted” list of uncultured protists. (a) Tree representing groups of approximately equivalent phylogenetic depth, based on the UniEuk taxonomy (Berney et al. 2017). Lineages are labeled in three possible colors: Dark purple, many representatives in culture (Parabasalia, Preaxostyla, Fornicata, Amoebozoa, Opisthokonta, Euglenozoa, Heterolobosea, Haptophyta, Rhizaria, Stramenopiles, Alveolata, Cryptophyceae, Chloroplastida, Rhodophyta); dark blue, few total species, but nearly all are in culture (Ancyromonadida, Malawimonadida, Anaeramoebae, Notopharyngea, Breviatea, Apusomonadida, *Mantamonas*, *Glissandra*, Rigifilida, Diphylleidae, *Tsukubamonas*, Jakobida, Provora, Caelestes, *Microheliella*, *Palpitomonas*, Glaucophyta, Rhodelphidia); red, few described species in culture or completely uncultured (Telonemia, Centroplasthelida, Hemimastigophora, Kathablepharidacea, Picozoa). The clade containing species that are currently within Provora, Caelestes, and Hemimastigophora has also been referred to as Promethea (Zlatogursky et al. 2025). Panels (b–j) indicate example groups currently lacking cultured representatives, whose cultivation could greatly add to knowledge of eukaryotic ecology and evolution: (b) Termite gut protists (Parabasalia): *Trichonympha agilis*, an anaerobic protist from the gut of the termite *Reticulitermes flavipes* . Scale bar 20 μm. (c) Arcellinids (Amoebozoa): *Difflugia* cf. *nodosa*, a member of Arcellinida with a symbiotic relationship with green algae (presumably Trebouxiophytes). These organisms mainly feed on other eukaryotes and need building material for their tests, and consequently are only possible to cultivate at present in highly mixed enrichment cultures. Scale bar 100 μm (image credit: Ferry Siemensma). (d) Eupelagonemids (Euglenozoa): No representative of the most abundant and diverse group of diplonemids (and important components of ocean plankton) is available in a stable culture, although short‐lived axenic cultures have been achieved. An image of an undescribed environmental eupelagonemid is shown. Scale bar 25 μm (image credit: Elizabeth Cooney). (e) Acantharia (Rhizaria): These protists produce inorganic skeletons and host photosynthetic eukaryotic symbionts, and are highly abundant in the open ocean. Scale bar 20 μm (image credit: Johan Decelle). (f) Collodarians (Rhizaria): These protists are also very abundant in the open ocean, and can form macroscopic syncytial colonies. Scale bar 1 mm (image credit: Johan Decelle). (g) MASTs (marine Stramenopiles): Two cells of MAST‐4 stramenopiles (green) on a particle (red with blue dots). MASTs are a diverse and abundant assemblage composed of 21 major clades that collectively represent a substantial proportion of the small heterotrophic eukaryotes in the upper ocean; unidentified members of MAST‐4 are among the most abundant (Obiol et al. 2024). Scale bar 5 μm (Piwosz et al. 2025) (reused under CC‐BY 4.0). (h) Syndiniales (Alveolata), a group of parasitic myzozoans with hundreds to thousands of distinct lineages based on 18S ribosomal RNA sequences. An image of the vermiform life history stage from an environmental sample. Scale bar 25 μm (image credit: Elizabeth Cooney). Cultivating members of this group would allow us to better understand their life cycles, host specificity, disease processes, and the evolutionary origins of parasitism. (i) *Picomonas judraskeda* (Picozoa), the only described member of this mysterious, diverse, and abundant lineage of heterotrophic flagellates, which was briefly isolated in culture and later lost. Scale bar 5 μm (Seenivasan et al. 2013) (reused under CC BY‐SA 4.0). (j) Environmental sequence surveys have identified numerous additional lineages that remain to be cultivated (Burki et al. 2021), many of which have uncertain phylogenetic positions within eukaryotes.

Much of our current understanding of protists derives from a long history of cultivation efforts (Box 1), which have enabled controlled investigations of cell biology, physiology, biochemistry, ecological interactions, and evolution. Many historically cultivated species were initially selected because of their impacts on human, animal, or crop health. However, only a limited fraction of protist diversity is currently maintained in laboratory culture. This cultivation gap hinders research on these important organisms and restricts our understanding not only of their biology and potential applications, but also of eukaryotic evolution and global biosphere functioning.

BOX 1 **A Brief History of Protist Culturing.**

The first observations of free‐living protists were made from environmental samples maintained in the laboratory by Leeuwenhoek (1677). Subsequent cultivation efforts relied on simple enrichment systems such as hay infusions (Kent 1880) and mixtures of organic nutrients (Beijerinck 1890). Early studies focused primarily on observing succession, reproduction, and morphology in ciliates and amoebae (Woodruff 1912), followed by attempts at continuous cultivation (Hyman 1925). One of the earliest axenic cultivations, that is, growth in the absence of other organisms, was achieved for *Euglena gracilis* (Dusi 1936).

During the first half of the twentieth century, many intensive cultivation efforts focused on protists pathogenic to humans and domesticated animals. One of the earliest successful examples involved the kinetoplastid *Trypanosoma brucei*, although only the insect‐associated procyclic stage could initially be cultivated (Novy and McNeal 1903); continuous cultivation of the bloodstream stage was achieved only nearly a century later (Brun et al. 1981). Stable xenic cultivation of the amoebozoan *Entamoeba histolytica* and cultivation of the ciliate *Balantidium coli* were reported in the 1920s (Barret and Yarbrough 1921; Barret and Smith 1924), whereas axenic cultivation of *E. histolytica* was achieved only decades later (Diamond 1961). Similarly, the diplomonad *Giardia intestinalis* was first cultivated together with yeast and chicken fibroblasts (Karapetyan 1962), followed by the establishment of axenic culture 8 years later (Meyer 1970). After numerous unsuccessful attempts to axenize the parabasalid *Trichomonas vaginalis* in order to test Koch's postulates (Hesseltine 1933), Trussell (Trussel 1940) eventually reported successful pure cultures. The history of cultivation of the malaria parasite *Plasmodium falciparum* proved particularly protracted and technically challenging, but ultimately culminated in continuous in vitro cultivation (Trager 1978).

The first systematic cultivation of photosynthetic protists (microalgae) is generally attributed to Martinus Willem Beijerinck, a pioneer of environmental microbiology. Beijerinck adapted bacterial cultivation techniques using gelatin‐based media to isolate and maintain algal strains, methods that later became highly influential in protistology. He additionally demonstrated that *Chlorella* sp. grew more efficiently when supplied with organic nutrients, revealing the capacity for mixotrophic metabolism (Beijerinck 1890). In the 1920s, Ernst Georg Pringsheim successfully isolated numerous algal strains and established one of the first dedicated centers for algal cultivation at Charles University in Prague. His pioneering work contributed substantially to the development of modern algal culture collections, including the Culture Collection of Algae and Protozoa (CCAP), the Sammlung von Algenkulturen Göttingen (SAG), the Culture Collection of Autotrophic Organisms (CCALA), and the Culture Collection of Algae at the University of Texas at Austin (UTEX) (see Supplementary Table 3 for a current list of worldwide culture collections).

Cultivation of non‐photosynthetic environmental protists also expanded during the late nineteenth and early twentieth centuries. Fritz Schaudinn maintained large protists such as foraminiferans and testate amoebae in culture and investigated patterns of cell and nuclear division (Schaudinn 1896). Interest in soil microorganisms and their influence on plant growth stimulated efforts to obtain “pure” or “monoprotistan” cultures. Cutler (Cutler 1923) cultivated *Naegleria* sp. and *Paracercomonas crassicauda* on sterile soil inoculated with bacteria in order to estimate the impact of protistan predation on bacterial populations, using serial dilution methods to obtain pure cultures. These early experiments helped establish the foundations of soil microbiome research and highlighted the ecological importance of protists in nutrient cycling and soil fertility (Ekelund and Rønn 1994). Short‐term cultivation approaches were also progressively developed to quantify protists in soils and freshwater environments (Cutler 1920; Darbyshire et al. 1974).

Together, these developments transformed protistology from a largely descriptive discipline into an experimental science and established many of the cultivation principles, methodologies, and infrastructures that continue to underpin modern protist research.

### 1.2 Protist Culturing to Unlock Eukaryotic Evolution and Ecology

Despite more than a century of cultivation efforts, cultured protists still represent only a small fraction of the environmental diversity revealed by more recent sequencing approaches (Pawlowski et al. 2012). Easily culturable species can be rare in their natural habitats (Lim et al. 1999; del Campo, Balagué, et al. 2013; Tashyreva et al. 2022; Valt et al. 2026), and cultivation efforts are strongly biased toward groups traditionally considered important for humanity. These limitations hamper our understanding of biological, ecological, and evolutionary processes. Nevertheless, the cultivation of novel protist species has enabled new discoveries in all of these fields (del Campo et al. 2024), including within groups such as amoebozoans and excavates that are commonly underreported or completely absent due to primer bias in metabarcoding studies.

Cultured protists have contributed to the discovery of both basic biological principles (Gibbons and Rowe 1965; Kruger et al. 1982; Greider and Blackburn 1985; Brownell et al. 1996) and exceptions from them, such as non‐canonical nucleotides and genetic codes (van Leeuwen et al. 1998; Kachale et al. 2023), the presence of unique compartments (e.g., glycosome, nitroplast) (Opperdoes and Borst 1977; Crowe and Morris 2021; Coale et al. 2024) or the absence of canonical ones (e.g., mitochondrion, Golgi apparatus) (Mowbrey and Dacks 2009; Karnkowska et al. 2016). Such examples clearly show that many major biological insights cannot be made simply by increasing environmental sequencing efforts. In addition, whereas prokaryotic genomes can often be assembled from metagenomic data into complete circular sequences, the large size of eukaryotic genomes, their high proportion of non‐coding DNA, and their organization into multiple linear chromosomes make this task considerably more challenging. Until recently, single‐cell genomics approaches have not provided data of sufficient quality, making cultivation‐based methods essentially the only way to obtain high‐quality genomic data for many eukaryotic lineages. Furthermore, cultivation provides access to rare organisms that would otherwise remain inaccessible using other approaches.

Cultivation enables direct experimental investigation of protist physiology, cell biology, genetics, behavior, and ecological interactions, as illustrated by numerous recent studies (Brunet et al. 2019; Hess et al. 2019; Hollender et al. 2024; Suzuki‐Tellier et al. 2024; Záhonová et al. 2025). Cultured protists also reveal extraordinary cellular and morphological diversity (Figure 2). Representative collections of cultured protists are also essential for interpreting environmental sequencing and metagenomic datasets by providing high‐quality reference genomes and validated biological context for sequence identification and annotation (Šimek et al. 2023).

**FIGURE 2:**
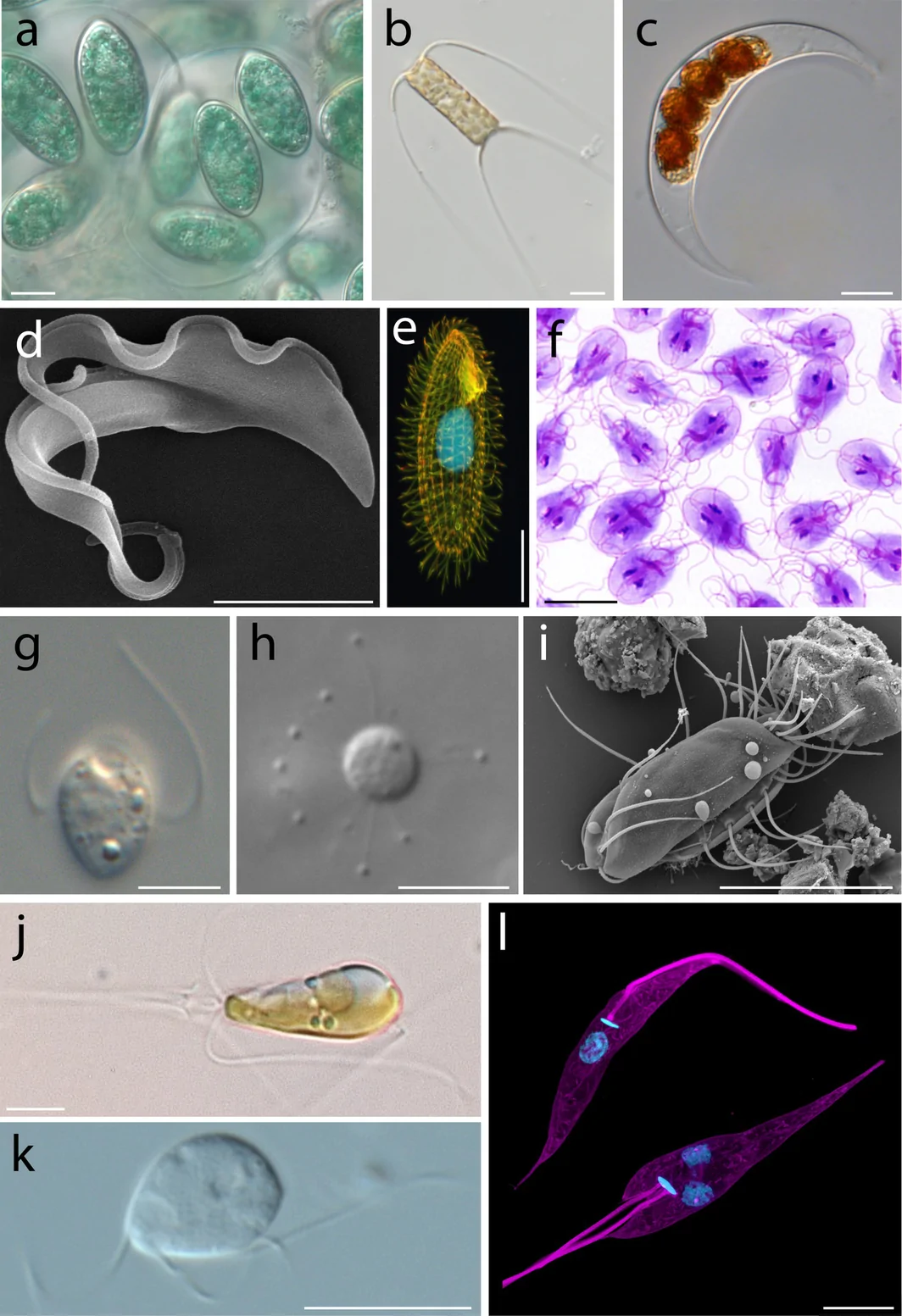
A survey of cultivated protist diversity. Lineage names in parentheses refer to Figure 1. (a) *Glaucocystis* sp. CCAP 229/2 (Glaucophyta), a glaucophyte alga. Scale bar 10 μm. (b) *Chaetoceros criophilus* CCAP 1010/55 (Stramenopiles), a centric diatom. Scale bar 10 μm. (c) *Dissodinium lunula* (Alveolata), a dinoflagellate that is a parasite of copepods. Scale bar 20 μm. (d) *Trypanosoma brucei* (Euglenozoa), a well‐studied parasitic kinetoplastid that is the cause of sleeping sickness in humans and animals. Scanning electron microscopy image of a dividing cell. Scale bar 10 μm. (e) *Tetrahymena thermophila* (Alveolata), whose cultivation led to the discovery of telomeres, catalytic RNA, histone acetylation, and numerous other features of eukaryotic biology. Scale bar 5 μm (Robinson 2006) (reused under CC‐BY 4.0). (f) *Giardia intestinalis* (Fornicata). Purified trophozoites of this well‐known intestinal parasite and model anaerobe, stained with Giemsa. Scale bar 10 μm (image credit: Břetislav Koudela, Veterinary University, Brno, Czechia). (g) An undescribed *Rhodelphis* strain (Rhodelphidia). Rhodelphids are recently discovered heterotrophic flagellates that, remarkably, are distantly related to red algae. Scale bar 5 μm (image credit: Elizabeth Weston). (h) *Solarion arienae* (Caelestes), a free‐living heterotrophic protist described in 2025 with sun‐like cells featuring multiple extrusome‐bearing stalks. Scale bar 5 μm (image credit: Marek Valt). (i) *Hemimastix* sp. (Hemimastigophora), a predatory multiflagellate with two sub‐membranous thecal plates. Hemimastigophora, together with Caelestes and other recently cultivated protist lineages, comprise a novel supergroup in the eukaryote tree (Figure 1a). Scale bar 10 μm (image credit: Elizabeth Weston). (j) *Braarudosphaera bigelowii* (Haptophyta), the first eukaryote with an organelle that can fix nitrogen (the nitroplast). This is one of a tiny number of independent cases where an endosymbiotic prokaryote has evolved dependency on extensive protein import from the host eukaryote, as in mitochondria and plastids. Scale bar 5 μm. (image credit: Wikipedia). (k) *Monocercomonoides exilis* (Preaxostyla), the first eukaryote shown to completely lack mitochondria and the proteins necessary for mitochondrial function. Scale bar 10 μm. (l) *Blastocrithidea nonstop* (Euglenozoa), a parasitic trypanosomatid whose genetic code has reassigned all three stop codons to encode amino acids, with only one of the three retaining a dual meaning as a stop signal (Kachale et al. 2023). Cells are expanded 4.5‐fold via expansion microscopy and stained with Sytox DNA dye (cyan) and NHS‐ester conjugated to Atto 595 (magenta), which stains amine groups and acts as a general protein marker. Scale bar 20 μm (image credit: Max Pendlebury, Biology Centre, České Budějovice, Czechia).

This review aims to provide an overview of protist cultivation, to foster renewed efforts toward the successful cultivation of diverse protist lineages. We discuss determinants of “culturability” to consider when attempting cultivation, and describe effective culturing strategies for some important ecological groups of protists. We also examine why some lineages have thus far proven recalcitrant to cultivation and highlight emerging innovations that could precipitate a “protist cultural renaissance” (del Campo et al. 2024).

## 2 A Practical Guide to Culturing Protists

A central principle of protist cultivation is to reproduce key aspects of the natural environment from which the organism was isolated. For free‐living protists, this includes the chemical and physical properties of the environment, together with suitable prey or microbial associates, where relevant. For parasitic or symbiotic protists, successful cultivation may require the presence of hosts or the simulation of host‐like conditions. Following isolation and identification from an environmental sample, numerous parameters can be adjusted in order to promote growth, induce specific physiological states, or trigger transitions between life cycle stages. These parameters can generally be grouped into four broad categories: (1) the growth vessel and incubation conditions, (2) the chemical and physical composition of the culture medium, (3) nutrient availability for the target species and its associated organisms, and (4) interactions with other organisms, as prey, hosts, symbionts, or microbiome partners.

### 2.1 How to Isolate and Identify Protists From Different Types of Samples

The first step in protist cultivation is the isolation of organisms of interest from the environment (Figure 3). Isolation strategies are often guided by the ecological and physiological characteristics of the target organisms. Isolating representatives of a specific taxonomic group can be more challenging than targeting a broad functional group. For instance, photoautotrophic protists can often be enriched by incubation under light conditions in an inorganic culture medium. In contrast, the isolation of previously uncultured taxa within a defined phylogenetic group may require prior knowledge of nutritional preferences, prey requirements, or environmental tolerances (Weber et al. 2017; Lyu et al. 2021).

**FIGURE 3:**
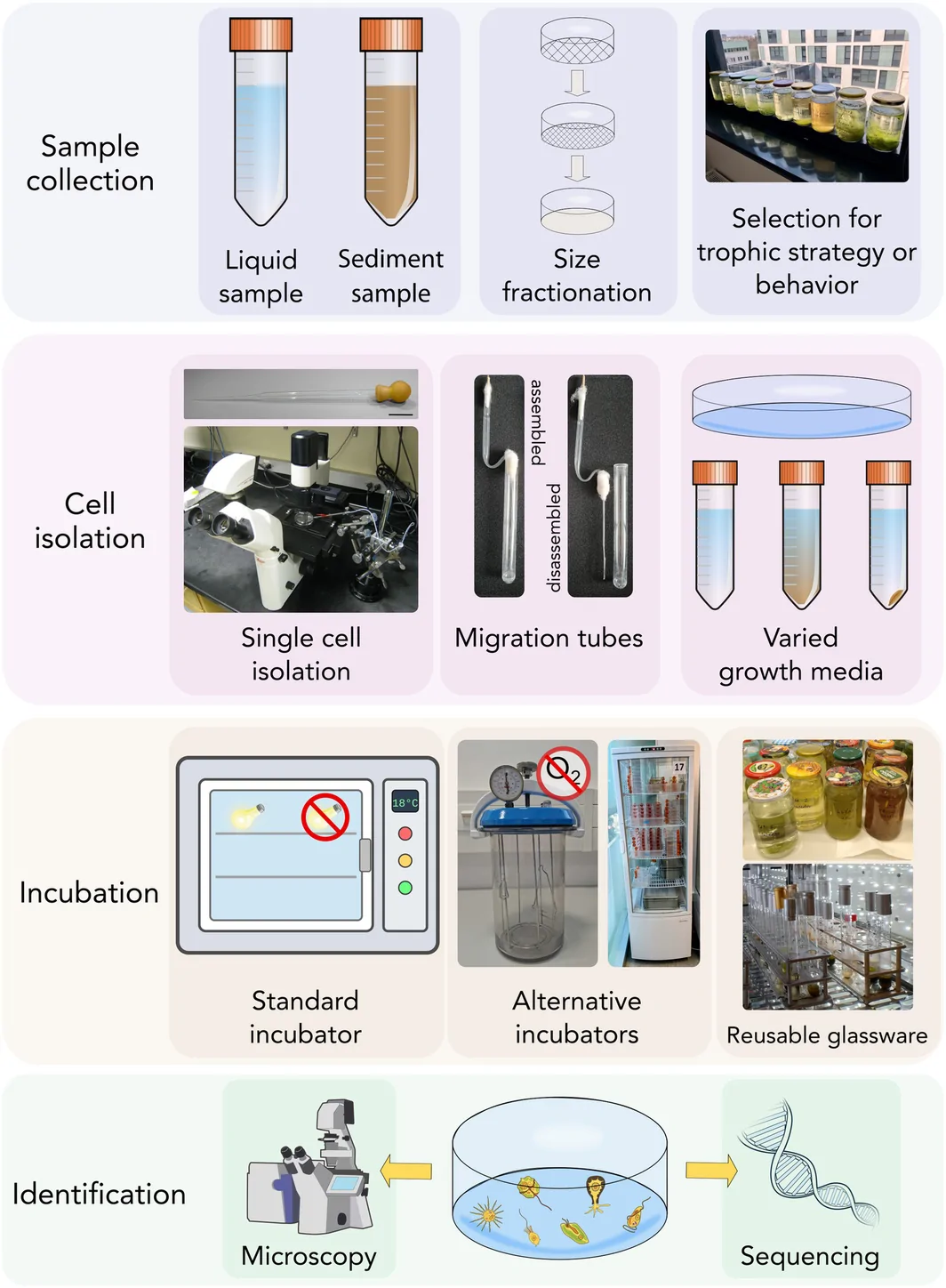
The protist culturing toolbox. Approaches from each step can be mixed and matched as necessary, depending on the target protist. We illustrate the most common approaches together with alternatives that could be more efficient or less costly. **Sample collection**: Samples can be collected in 50 mL conical tubes (clip art from (De Oliveira 2022)), or larger volumes of liters (or more) can be collected if environmental cell density is low or to maintain temperature during transport. Pelagic samples can be concentrated with plankton nets (not shown). Attached protists can be collected by scraping the edge of the collection vessel on a substrate. Size fractionation can be performed, either to enrich for targeted protists of a known size, or to eliminate unwanted species (e.g., larger predators or bacteria). Vessels with mixed communities can be enriched prior to isolation; for example, they can be placed near light to select for phototactic organisms. **Cell isolation**: If the target protist is abundant in the natural sample, serial dilution can be used for isolation. Protists can also be isolated by single‐cell picking. For larger protists, a pasteur pipette can be used (image credit: Carlos de Mier; scale bar 2 cm). For smaller protists, the example setup shown here uses a pulled glass pipette, a Narishige micromanipulator, and the sample in a Petri dish on an inverted microscope. Alternatively, if motile protists are targeted, they can be isolated from non‐motile protists via migration tubes (on the left, an assembled migration tube; on the right, a pre‐assembly migration tube). Cell isolation is performed into different types of growth media in order to increase the chances of cell growth: For example, liquid media in Petri dishes or tubes, tubes with biphasic medium, or tubes with cereal grains as a nutrient source (Petri dish clip art from (Carpaneto 2021)). **Incubation and growth**: Incubation of protists, typically in an incubator that can control temperature, humidity, light, and other conditions, for a period of days to weeks, depending on the growth rate of the target protist. Alternatively, for anaerobic or microaerophilic cultivation, specialized anaerostat chambers can be used. When natural light is required but temperature must be controlled, refrigerated pastry display cases or simply leaving growth vessels at room temperature near a window can be an effective, economical option. To encourage reusability and reduce expenses, glass jars and reusable glassware can be used in place of single‐use plastic flasks. **Identification**: Species identification of the isolated protists with a combination of morphological characters via various types of microscopy (DIC, phase contrast, fluorescence, EM) and sequencing, typically of standard markers such as the ribosomal RNA gene locus (microscope and DNA illustrations from NIAID NIH BioArt, IDs 000086 and 000124; protist drawings from (Keeling and Eglit 2023)).

Initial enrichment can be facilitated by preparing a wide variety of amended and unamended media based on natural water, to which the environmental sample is added (Lee 1993). Amendments may include inorganic nutrients for photoautotrophic species, dissolved organic substrates for osmotrophic taxa or those feeding on heterotrophic bacteria, or prey organisms (typically live) for phagotrophic protists. If the target species falls within a known size fraction, enrichment can also be achieved through selective filtration. Size‐fractionated and/or amended samples can then be incubated for periods ranging from days to weeks, allowing target organisms to proliferate in the reduced presence of predators or competitors (Massana et al. 2006; King et al. 2009; Piwosz and Pernthaler 2011; Grujčić et al. 2015). Benthic protists can be separated from sediment using density gradient centrifugation (Price et al. 1978; Alongi 1993), by incubating coverslips on sediment preparations, separated by a porous paper barrier (Larsen and Patterson 1990), or by using the seawater‐ice elution (“Uhlig”) method, in which a layer of seawater ice placed above the sediment creates a descending cold and oxygenated front that stimulates organisms to migrate into the underlying seawater for collection (Uhlig et al. 1973). The choice of liquid versus solid media can also exert a strong effect on the species that are initially enriched (see Section 2.3).

Following enrichment, clonal cultures can be established using a range of isolation techniques. Serial dilution of enriched inocula in sterile medium is particularly effective for pico‐ and nano‐sized protists (< 20 μm) (Caron 1993; King et al. 2009). The number of dilution steps required depends on the abundance of the target organisms in the inoculum, which must be determined in advance. Larger protists (> 20 μm) can often be isolated individually using micropipettes followed by successive washing steps to reduce contamination (Pringsheim 1946; Droop 1954). Isolation of individual nano‐ or even pico‐sized protists using this approach is technically possible, but more challenging (e.g., (Nitsche et al. 2017; Eglit et al. 2024; Zlatogursky et al. 2025)). High‐throughput isolation approaches increasingly rely on Fluorescence‐Activated Cell Sorting (FACS) or Image‐Activated Cell Sorting, which enable the identification, selection, and isolation of target cells based on optical or morphological properties (del Campo, Not, et al. 2013; Schraivogel et al. 2022; Kuhn et al. 2024). Additional methods have been developed for particular protist groups. For example, clonal cultures of ciliates have been obtained using the silicone oil plating procedure (Soldo and Brickson 1993). Isolation may also be facilitated by exploiting characteristic protistan behaviors such as phototaxis, geotaxis (Soldo and Brickson 1991), galvanotaxis (Kišidayová and Váradyová 2004), magnetotaxis (Chen et al. 2021) or predation on magnetotactic bacteria (Monteil et al. 2018), all of which can help concentrate target cells prior to single‐cell isolation.

Following isolation, cultures are typically identified using complementary microscopic and molecular approaches. Light microscopy, particularly with phase‐contrast or differential interference contrast optics, remains essential for identifying cell morphology, motility, ciliary or flagellar structures, pseudopodia, chloroplast organization, and cell wall and extracellular structures such as loricae, thecae, or frustules (see Box 2: Recommended Literature for a list of guides to protist identification). Electron microscopy provides additional ultrastructural information at nanometer‐scale resolution, including details of scales, pores, spines, membranes, and organelles.

BOX 2 **Recommended Literature.**

**Culturing methods**

1. A comprehensive collection of methods in aquatic microbial ecology, with step‐by‐step instructions and practical advice (Kemp et al. 2018).
2. A thorough review of laboratory equipment, sterilization, illumination, isolation, media recipes, and culture maintenance. Written specifically for harmful marine microalgae, but the techniques described are more generally applicable (Guillard and Morton 2004).
3. Protocols in Protozoology, published in 1992 by the Society of Protistologists (Lee and Soldo 1992).
4. A book covering aspects of isolation, purification, growing, and working with photosynthetic protists (Phycological Society of America and Andersen 2009).

**Protist identification**

1. A taxon‐by‐taxon guide of 43 eukaryotic groups, each accompanied by a summary of specific literature, habitats and ecology, morphology and ultrastructure, life history, cultivation and systematics (Archibald et al. 2017).
2. A fully illustrated guide to 45 eukaryotic groups, with a general key for distinguishing among groups and key characters for species identification within each group (Lee et al. 2000).
3. A short guide for morphogroupings of flagellates (Jeuck and Arndt 2013).
4. A visual guide for initial morphogroupings of amoebozoan amoebae (Smirnov et al. 2011).
5. An illustrated guide for the identification of all major autotrophic and heterotrophic protist groups found in freshwater environments (Patterson and Hedley 1996).
6. An extensive guide providing taxonomy and ecological background on all freshwater algae, including both unicellular and multicellular species (Wehr et al. 2015).
7. A guide to 300 ciliate species used as bioindicators (Foissner and Berger 1996).
8. A website that includes images of many amoeboid organisms: https://arcella.nl/ (Ferry Siemensma).

**Selected recent triumphs in culturing**

1. *Tyrannomonas regina* (Cryptophyceae): one of the key bacterivores in freshwater lakes, and the first cultured representative of the abundant and diverse CRY1a lineage (Mukherjee et al. 2025).
2. *Braarudosphaera bigelowii* (Haptophyta), a photosynthetic protist that is the first known eukaryote with an organelle that can fix nitrogen (the nitroplast); much of the characterization of this system relied on cultivation of *B. bigelowii* (Coale et al. 2024).
3. *Meteora sporadica* (Caelestes): a protist with a unique morphology including a pair of swinging lateral “arms” that was independently re‐isolated and cultured (Galindo et al. 2022; Eglit et al. 2024) after its initial observation 20 years earlier (Hausmann et al. 2002).
4. *Solarion arienae* (Caelestes): another protist with a unique and curious morphology, whose cultivation and sequencing led to a phylogenetic placement sister to *Meteora* within the newly created Disparia (Valt et al. 2026).
5. *Choanoeca flexa* (Opisthokonta): a thrilling demonstration of how ecological and evolutionary hypotheses, in this case on colony formation in the closest relatives of animals, can be formulated based on experiments performed on a recently‐isolated culture, and then tested in situ at the original location where the culture was isolated (Ros‐Rocher et al. 2026).

Molecular identification is generally based on amplification and sequencing of marker loci from extracted DNA. The most widely used markers are regions of the ribosomal operon, including the 18S, 5.8S, and 28S rRNA genes, together with the ITS regions. Mitochondrial markers such as cytochrome c oxidase I (cox1) and other loci may also be informative for particular groups. Comparison of sequences against curated reference databases can then provide taxonomic identification, with the level of resolution dependent on the taxonomic breadth and quality of the available databases (Guillou et al. 2012; Berney et al. 2022; González‐Miguéns et al. 2025). These comparisons are generally complemented by phylogenetics, especially if the new isolate lacks close relatives.

### 2.2 Growth Vessels and Incubation Conditions

One of the first practical considerations in protist cultivation is the choice of an appropriate growth vessel. Commonly used vessels include angled‐neck flasks, Erlenmeyer flasks, glass tubes, and conical tubes. These are generally transparent, which permits light transmission and in some cases allows observation by inverted microscopy. Growth vessels are typically made of either plastic or glass and some species may preferentially grow in one material or the other (Stein and Hellebust 1979). In light of the fact that they are single‐use, angled‐neck flasks are typically more costly, but are more convenient for microscopy and routine handling. The choice of flask also depends on the morphology, behavior, and life cycle of the organism being cultivated. Some protists, including most amoebae, some flagellates, and ciliates, and many diatoms, preferentially attach to surfaces or crawl, glide, or skid along substrates. Others float freely in the water column or swim through the medium (most algae, many flagellates, and ciliates) and may have specific preferences for particular medium depths or surface‐area‐to‐volume ratios (Wu et al. 2024). Biofilm formation and the development of bacterial films on vessel surfaces may also strongly influence growth, particularly for benthic, or surface‐associated taxa.

Perhaps the most influential external condition to be controlled is temperature, which has been shown not only to impact growth rate but also numerous other aspects of protist physiology (Rose et al. 2009; Edwards et al. 2016; Franzè and Menden‐Deuer 2020). Growth temperature is typically controlled by a laboratory incubator, although many species tolerate ambient room temperatures during routine maintenance. Importantly, conditions that maximize growth are not always optimal for long‐term maintenance, and lower temperatures are often used to slow division rates and reduce transfer frequency, particularly for strains that cannot be cryopreserved (see Section 2.7).

The most common laboratory incubator temperatures range between 10°C and 37°C, although the diversity of protists encompasses much broader thermal tolerances. For parasitic protists, the temperature may vary substantially depending on life cycle stage, such as in the case of the kinetoplastid *Trypanosoma brucei* for which the insect‐transmitted and mammalian‐infective stages grow at 27°C and 37°C, respectively (Kaminsky et al. 1988). For polar species, such as the sea ice diatom *Haslea vitrea*, temperature may be maintained as low as −1°C (Brown et al. 2020), whereas the amoebozoan *Incendiamoeba cascadensis* can successfully undergo division at temperatures up to 63°C (Rappaport et al. 2026).

If the target species or its obligate prey or partners are photosynthetic, natural, or artificial light must also be provided. Conversely, non‐photosynthetic cultures are often maintained in the dark to discourage photosynthetic contaminants, or to prevent predators from ingesting potentially toxic photosynthetic byproducts (Kashiyama et al. 2012, 2019). Natural daylight can be used to mimic seasonal variation and may be sufficient when cultures are maintained near windows or in incubators with transparent walls. Artificial light is generally supplied by fluorescent or LED sources, with modern cultivation systems increasingly relying on LED lighting because of its stability, low heat production, energy efficiency, and ease of controlling spectral composition and intensity. Both light intensity and photoperiod should be adapted to the ecological requirements of the organisms being cultivated. Excessively low irradiance may limit growth, whereas overly intense illumination can damage light‐sensitive taxa (including some green algae, diatoms, and euglenids). Common light intensities range from 100 to 400 μmol photons m^−2^ s^−1^ (Mantzorou and Ververidis 2019), however, some algal taxa, for example those growing beneath sea ice, have very low light requirements (< 0.17 μmol photons m^−2^ s^−1^) (Hancke et al. 2018), whereas others isolated from environments with high irradiation such as deserts can tolerate intense light (up to 2000 μmol photons m^−2^ s^−1^) (Levin et al. 2024). Common diel photoperiods include cycles of 8–16 h of light followed by 16–8 h of darkness, although continuous illumination and other customized light regimes are also frequently employed depending on experimental objectives (Chisholm et al. 1975; Legrand et al. 1998; Moore et al. 2008; Foflonker et al. 2016).

Protists with low‐oxygen or anaerobic lifestyles require additional control of atmospheric conditions. Many free‐living anaerobes can be maintained simply by growing their (usually mixed) prokaryotic prey at high density in nutrient‐rich media within nearly full vessels (i.e., with minimal headspace). Under these conditions, microbial respiration rapidly depletes dissolved oxygen in the medium, while anaerobic protists often migrate to the bottom of the vessel, well away from the air‐water interface (Kolisko et al. 2010) Other species require their growth medium to be deoxygenated by bubbling with CO_2_ (also referred to as sparging or insufflation in the literature) or the headspace to be replaced with argon (Stabel et al. 2021; Aguilera‐Campos et al. 2025). In these cases, resazurin can be employed as a convenient aerobic/redox indicator (Karakashev et al. 2003). Conversely, strictly aerobic species may require enhanced gas exchange. These organisms are often maintained in vessels fitted with gas‐permeable filter caps that permit oxygen diffusion while limiting contamination. Species with high oxygen demand may additionally require agitation or aeration to prevent oxygen depletion, particularly in vessels with low surface‐area‐to‐volume ratios. Agitation can also be used to promote specific life history transitions, such as multicellular aggregation in the filasterean *Capsaspora owczarzaki* (Sebé‐Pedrós et al. 2013).

Protists isolated from high‐pressure environments such as the deep ocean frequently tolerate ambient atmospheric pressure during laboratory cultivation (Atkins et al. 1998; Tashyreva et al. 2022), whereas others require simulated high‐pressure conditions to grow successfully (Turley et al. 1988). Specialized growth conditions can also be achieved through the use of custom‐designed microfluidic chambers. These systems allow precise control of environmental parameters such as flow, oxygen gradients, and chemical composition, as recently illustrated by the flow‐through cultivation system developed to culture the ciliate *Zoothamnium niveum* and its sulfide‐oxidizing prokaryotic symbionts (Contarini et al. 2024).

### 2.3 Chemical and Physical Composition of the Culture Medium

The choice of liquid versus solid growth medium may exert a strong influence on protist growth in culture. Some species can grow successfully in both liquid and solid media (e.g., the euglenozoan *Euglena gracilis* , the heterolobosean *Naegleria gruberi* , and the amoebozoans *Acanthamoeba* spp.). Others show clear preferences for either liquid media, as is common among free‐swimming protists, or solid substrates, which frequently promote the growth and reproduction of amoeboid taxa. In addition, some protists preferentially grow at solid–liquid interfaces in biphasic media. This is the case for the kinetoplastid *Trypanoplasma borreli*, as well as various parabasalids, oxymonads, and retortamonads (Lukes et al. 1994; Cepicka et al. 2006; Treitli et al. 2018).

The choice between liquid and solid media also depends on downstream applications. Solid growth media allow dilute cultures to form macroscopic clonal colonies that can be readily isolated and transferred. Such colonies can be used to establish clonal protist cultures, isolate bacterial prey strains for subsequent co‐culture, or perform experimental manipulations. In myxomycetes, agar‐based media supplemented with oat grains are commonly used to obtain plasmodia from environmental samples, as the developing plasmodia feed on bacteria growing on the substrate (Chen et al. 2013). Conversely, nutrient‐rich liquid media are often preferred for maintaining axenic cultures of model organisms such as the amoebozoan *Physarum polycephalum* (Daniel and Baldwin 1964). In oomycetes such as *Saprolegnia*, solid media amended with antibiotics are used to obtain bacteria‐free hyphae (Seymour 1970).

The chemical composition of the culture medium is another major determinant of cultivation success. Salinity is often adjusted to reflect the environmental conditions from which the organisms were isolated and may range from freshwater conditions to extreme hypersalinity. For example, the heteroloboseids *Tulamoeba peronaphora* and *Pleurostomum flabellatum* tolerate salinities of 75‰–250‰ and 200‰–300‰, respectively (Park et al. 2007, 2009). Many protists are nevertheless euryhaline and can grow across broad salinity ranges that differ substantially from those of their original habitats (Schiwitza et al. 2018; Useros et al. 2023).

Similarly, the pH of the culture medium is generally adjusted to match the original environmental conditions, for example those from hyperalkaline lakes (Gigeroff et al. 2023; Hake et al. 2024). pH strongly influences carbonate system equilibria and the chemical speciation and bioavailability of trace metals in culture media, thereby affecting inorganic carbon availability, nutrient uptake, and potential metal toxicity. For growth media lacking an intrinsic buffering capacity, chemical buffers can be added to prevent large changes in pH, although the growth of some species may be inhibited by the buffering agent itself, for example TRIS for dinoflagellates (Guillard and Morton 2004) and HEPES for the choanoflagellate *Codosiga hollandica* (Richter et al. 2018). In these cases, sparging with CO_2_ followed by flushing of excess CO_2_ with air can be used to prepare buffered media prior to growth (Connon and Giovannoni 2002). Antioxidants, such as ascorbic acid, are occasionally added to culture media for anaerobic protists to mitigate oxidative stress by scavenging reactive oxygen species. Growth media can be prepared using either environmentally derived or artificial base solutions, balancing ecological realism, reproducibility, and control over chemical composition. One common and convenient approach is to use sterilized natural water collected from the source environment, such as seawater, lake water, or pore water. This facilitates reproduction of the original physicochemical conditions and may provide undefined growth‐promoting compounds, dissolved organic matter, or microbial metabolites that support the cultivation of fastidious taxa. However, natural water‐based media are chemically incompletely defined and may vary spatially, seasonally, or between sampling events, potentially reducing reproducibility among experiments and cultures. For marine media, commercially available aquarium salt mixes can be used as convenient artificial seawater bases. These mixes are inexpensive, easy to prepare, and generally suitable for maintaining many marine protists. However, their precise composition is often proprietary or optimized for aquarium animals rather than microbial cultivation. Consequently, aquarium salts may be useful for routine cultivation and enrichment of many (although not necessarily all) protist species, but may be less suitable for physiological experiments requiring tightly controlled chemical conditions.

Artificial solutions provide greater chemical control and reproducibility. In these media, purified water (i.e., distilled or Milli‐Q water) is supplemented with major ions (e.g., Na^+^, Cl^−^, Mg^2+^, Ca^2+^, K^+^, SO_4_^2−^), trace metals, vitamins, and other components in defined proportions intended to reproduce the osmotic and chemical characteristics of the original environment. Major nutrients such as nitrogen and phosphorus must also typically be added, commonly in the form of nitrate, ammonium, phosphate, or organic nutrient sources depending on the trophic strategy of the target organisms. Trace elements such as Fe, Cu, Mo, Zn Co, Mn, Se, and Ni may additionally be required, although their bioavailability is often more important than their absolute concentration. Chelating agents such as EDTA are therefore commonly added to maintain metals in bioavailable forms (Guillard and Morton 2004). Vitamins and other growth factors may also be included depending on the requirements of the cultured organisms. Numerous standardized formulations based on these principles are available (Supplementary Tables 1 and 2), reducing the need for investigators to develop media recipes *de novo*.

Before use, culture media must be sterilized. Common sterilization methods include filtration through small pore size (≤ 0.22 μm) membranes, autoclaving, and microwave sterilization. Each method has advantages and limitations. Filtration preserves thermolabile compounds but may not eliminate all small bacteria or viruses from the medium. Autoclaving is widely regarded as the most reliable sterilization method and has been described as “the ultimate guarantee of sterility” (Guillard and Morton 2004). However, it must be used carefully, as heating causes CO_2_ release and thus elevated pH levels that can lead to the formation of precipitates which may inhibit growth or reduce nutrient availability (Guillard and Morton 2004). Precipitation problems can sometimes be mitigated by lowering pH prior to autoclaving and restoring pH levels with sterile NaOH after autoclaving, or by an additional filtration step. In addition, the thermolabile components of the medium (notably vitamins) may be destroyed by autoclaving and must instead be sterilized separately and added after cooling. Microwaving has been shown to be effective for sterilization (Keller et al. 1988; Price et al. 1989), although its use is constrained by vessel size and material compatibility.

Specific nutrient requirements depending on trophic strategy are discussed in the following section. For protists that depend on prey, symbionts, or other microbial associates, the nutritional and physiological requirements of the entire consortium must be considered when designing growth media (see Section 2.6).

### 2.4 Trophic Strategies and Nutrient Requirements

Successful protist cultivation depends strongly on the trophic strategies of the target organisms, which determine their nutritional requirements and interactions with the surrounding microbial community. Because protists occupy virtually all ecological niches, they exhibit an extraordinary diversity of metabolic strategies and nutrient acquisition mechanisms. Broad trophic categories include phototrophy, osmotrophy (including saprotrophy), and phagotrophy, although these strategies are not mutually exclusive and may vary across life cycle stages or environmental conditions.

Many protists are mixotrophic and can combine multiple trophic modes simultaneously or sequentially, for example through combinations of photosynthesis, uptake of dissolved organic compounds, and ingestion of prey organisms. Consequently, successful cultivation often requires not only appropriate chemical nutrients but also suitable prey, symbiotic partners, organic substrates, vitamins, or environmental conditions tailored to the physiology of the target species.

#### 2.4.1 Saprotrophs, Osmotrophs, and Phototrophs

Many protists do not depend on live prey or hosts and instead acquire nutrients directly from dissolved compounds or decomposing organic matter in their environment (Geisen et al. 2018). Osmotrophic protists absorb dissolved organic and inorganic compounds across the cell membrane, whereas saprotrophy represents a specialized form of osmotrophy in which extracellular enzymes degrade complex organic material into soluble compounds that can subsequently be absorbed. These trophic strategies are widespread across the eukaryotic tree of life and often occur alongside other nutritional modes.

Fungi are among the best‐known saprotrophic eukaryotes, but many protists also contribute substantially to decomposition processes. For example, the myxomycete *Badhamia polycephala* (previously *Physarum polycephalum* ) is capable of degrading chitin through extracellular chitinases (Renaud et al. 2023), although it is also strongly bacterivorous. In contrast, the labyrinthulid *Aplanochytrium* lacks phagocytic capacity and relies exclusively on lysotrophic saprotrophy for nutrient acquisition (Hamamoto and Honda 2019). Facultative saprotrophy is widespread among protists and has been documented across many major eukaryotic clades (Maillard et al. 2025).

Many saprotrophic and osmotrophic protists are relatively straightforward to cultivate because their natural substrates can often be incorporated directly into culture media after sterilization. Agar‐based media are particularly widely used and reproducible for these organisms. For example, labyrinthulomycete thalli can be readily propagated on agar supplemented with diverse nutrient sources (Pereboev and Bubnova 2023). Depending on the target species, media may additionally be supplemented with specific carbon sources, amino acids, vitamins, or complex organic substrates that mimic natural decomposition environments.

Phototrophic protists, commonly referred to as microalgae, include numerous obligately or facultatively phototrophic lineages distributed across diverse major lineages (Jassey et al. 2022). These organisms use photosynthesis to fix inorganic carbon into organic compounds and therefore require light, CO_2_, and appropriate inorganic nutrients for growth. Nutrient requirements vary substantially among species and may include nitrogen sources such as nitrate or ammonium (particularly in taxa lacking nitrate reductase) (Guillard and Morton 2004), phosphorus, trace metals such as iron, and vitamins including B_12_. Some nutrients are lineage‐specific; for example, silica is essential for frustule formation in diatoms (Leontieva and Kirpenko 2020).

Many phototrophic protists are also metabolically flexible and may additionally utilize dissolved organic compounds or prey organisms under particular environmental conditions. Furthermore, some species depend indirectly on associated bacteria that provide essential vitamins or growth‐promoting compounds. Consequently, successful cultivation often requires careful adjustment not only of light conditions but also of nutrient composition and, in some cases, microbial context (see Section 2.6).

Despite the broad diversity of phototrophic protists and their nutritional requirements, a relatively small number of standardized media formulations (Supplementary Table 1) have successfully supported the cultivation of a wide range of taxa, even though these formulations may not necessarily be optimal for any individual species.

#### 2.4.2 Phagotrophs (Predators of Bacteria and Eukaryotes)

Although phototrophic protists can numerically dominate aquatic plankton communities, predatory protists represent the largest component of global protist functional diversity (Oliverio et al. 2020; Singer et al. 2021). Most of these consumers are generalist feeders capable of exploiting a wide range of prey types rather than specializing in a single taxon (Geisen et al. 2023). Nonetheless, prey size is a very important factor in determining feeding capacity. Many protists are restricted to bacterivory because they cannot efficiently ingest larger prey such as fungi or algae (Singer et al. 2021); metabolic requirements and the ability to subdue potential prey may also play a role. Prey identity and nutritional quality can additionally influence key growth parameters of the predator, including doubling time, growth efficiency, lag phase duration, and overall culture stability (Grujčić et al. 2015).

Successful cultivation of phagotrophic protists often depends as much on maintaining suitable prey populations as on the physiology of the target organism itself. Many consumers can be cultivated together with their co‐isolated prey in liquid or agar medium designed to support growth of the prey (Smirnov and Brown 2004). Widely used enrichment media include cereal grass infusions, direct addition of autoclaved grains such as wheat, barley, rice, buckwheat, or quinoa, or preparations of organic material such as yeast extract or proteose peptone (Supplementary Table 1). These substrates promote bacterial or fungal growth that subsequently supports predatory protists.

Alternatively, standardized prey organisms can be added directly to cultures. Common bacterial prey include *Escherichia coli* and *Klebsiella (Enterobacter) aerogenes*, while yeast or readily cultivated protists are often used for eukaryovorous species. Prey may be supplied either alive or heat‐killed, or they may be pre‐grown prior to inoculation with the predator (Keenan et al. 1978; Tikhonenkov et al. 2022). In some cases, predators exhibit strong prey preferences or grow optimally only with particular prey taxa, indicating that cultivation efforts may require testing multiple prey organisms and media formulations in parallel (Mrva and Garajová 2018). Long‐term maintenance of predator–prey systems may also require balancing prey growth and grazing pressure in order to avoid collapse of either population.

Successful cultivation therefore may require optimization of conditions for both predator and prey, including nutrient composition, trace metals, vitamin availability, and physical growth conditions. In many cases, media requirements are determined not only by the consumer itself but also by the physiological demands of its prey organisms (Wanner et al. 2016).

### 2.5 Culturing Parasites

Obligate parasites are thought to constitute a major fraction of global protist species richness and can strongly influence the biology, ecology, and evolution of their hosts (Geisen et al. 2018; Anthony et al. 2023). It has been suggested that nearly all animal species, including the hyperdiverse insects, host at least one species‐specific parasitic protist (Mahé et al. 2017). This suggests that many parasitic protists depend on host species‐specific nutrients, metabolites, environmental conditions, or developmental signals for survival and proliferation.

Among these organisms, obligate intracellular parasites are arguably the most difficult group of protists to cultivate. As a result, the most reliable strategy for cultivating obligate intracellular parasites is often to maintain them in association with their original hosts or with suitable host‐derived cells or tissues. For well‐characterized protist parasites, established host cell systems can be used for in vitro propagation. For example, the apicomplexan *Plasmodium* can be grown using erythrocyte cultures. Modern cultivation approaches increasingly rely on immortalized cell lines or other host‐derived culture systems that reproduce key physiological characteristics of the host environment.

In contrast, parasites occupying extracellular host niches, such as luminal or blood plasma environments, are often comparatively easier to cultivate because they frequently can be maintained in media designed to mimic host‐like physio‐chemical conditions. This applies to organisms such as trypanosomes, trichomonads, *Entamoeba*, and *Giardia*, which often can be cultivated axenically (Diamond 1957; Diamond et al. 1978; Keister 1983) or in association with bacteria (Dobell and Laidlaw 1926). The specific medium to be used does not necessarily need to be known a priori. For example, trypanosomatids isolated from insects are commonly transferred immediately into several different growth media in parallel, increasing the probability that at least one medium will support successful establishment and long‐term maintenance of the isolate (Lukeš and Votýpka 2020).

Facultative parasites, or those with a free‐living stage, are easier to cultivate than obligate parasites. For example, *Naegleria fowleri* and *Acanthamoeba* spp., potential human pathogens that are largely free‐living, can be cultured on agar or in liquid conditions, either with bacteria or axenically with a specific nutrient mixture (Schuster 2002).

### 2.6 Eliminating or Reducing Unwanted Culture Partners

Protists isolated from natural environments frequently coexist with other microorganisms, including prey organisms, symbionts, commensals, parasites, or opportunistic contaminants. In many cases, these associated organisms initially persist in laboratory cultures and may even be required for successful growth of the target protist. Nevertheless, a major objective of many cultivation efforts is to obtain cultures dominated by a single protist strain, ideally at high density and with minimal presence of other microorganisms. This improves standardization and reproducibility of experiments and simplifies interpretation of physiological, ecological, and genomic data (Altermatt et al. 2015). Contaminating organisms may additionally interfere with microscopic observations and complicate downstream applications such as genome sequencing or transcriptomics. Cultivation therefore often involves balancing experimental simplicity and reproducibility against ecological realism and biological viability.

Many cultivation efforts aim to establish axenic cultures, in which the target protist is maintained in the absence of all other living organisms. Achieving axenic cultures is often challenging because associated prokaryotes or eukaryotes must be eliminated without compromising the viability of the protist itself (and the contaminating species may offer differing levels of resistance to elimination). Common approaches include treatment with antibiotics, mechanical filtration, serial dilution, and isolation of single cells (Lagkouvardos et al. 2014; Vu et al. 2018; Prokopchuk et al. 2019; Mei et al. 2023). Motile protists can additionally be separated from non‐motile contaminants such as yeast using migratory U‐shaped tubes, while S‐shaped variants are used for anaerobic taxa (Lukeš and Votýpka 2020). Although axenization can be technically demanding, it has proven feasible for many algae and parasitic protists once suitable cultivation conditions are identified.

Axenization becomes substantially more difficult for bacterivorous and eukaryovorous protists that depend on prey organisms for survival. Successful establishment of axenic cultures in these cases requires development of nutrient sources capable of replacing live prey and supporting both growth and essential cellular functions (Droop 1970; Skelton et al. 2009). This has nevertheless been achieved for some facultatively predatory protists, including *Acanthamoeba* spp. and the ciliate *Tetrahymena thermophila*, which can supplement phagotrophy through osmotrophic nutrient uptake (Penland and Wilhelmus 1997; Cassidy‐Hanley 2012).

For many protists, however, complete axenization may neither be feasible nor biologically appropriate. Numerous species naturally depend on bacteria or other eukaryotes as prey, metabolic partners, or providers of essential nutrients and growth factors. In these situations, co‐cultivation can create more ecologically realistic conditions that better reflect the natural biology of the organism. One widely used compromise is monoxenic cultivation, in which the target protist is maintained together with a single defined bacterial or eukaryotic species. This approach preserves key ecological interactions while substantially simplifying downstream analyses such as genome sequencing and metabolic studies (King et al. 2008; Fairclough et al. 2013; Akdeniz et al. 2025). However, identifying suitable prey or partner organisms can be difficult because many protists exhibit selective feeding behaviors and complex nutritional dependencies; an added difficulty is that prey that may be easy to culture may not necessarily be adequate food sources (e.g., *S. cerevisiae* lab strains). As a result prey identity may strongly influence growth dynamics, physiology, and experimental outcomes (Florenza and Bertilsson 2023). Defined multispecies systems or synthetic microbial communities may therefore represent promising future approaches for reconstructing ecologically relevant but experimentally tractable cultivation systems.

Some protists additionally maintain obligate or highly stable symbiotic relationships with ecto‐ or endosymbionts, making removal of associated organisms difficult or impossible without compromising host survival. In these cases, a holobiont perspective, that is, studying the protist together with its associated symbiotic community, may be more appropriate for understanding physiology, metabolism, and ecological function. Examples include photosymbioses and kleptoplasty (Decelle et al. 2015), as well as associations with bacterial endosymbionts whose functions may be partially or entirely unknown (George et al. 2020; Husnik et al. 2021). Kleptoplastidic protists provide a particularly striking example because they depend on continual acquisition of chloroplasts from prey organisms in order to maintain photosynthetic capacity (Gast et al. 2007; Johnson et al. 2023; Karnkowska et al. 2023). Ideally, all essential partners within such systems should be identified and maintained together under controlled co‐culture conditions.

In many cases, however, the complete taxonomic composition of the culture remains unresolved, resulting in xenic cultures in which multiple unidentified organisms coexist. Although more difficult to standardize and interpret, xenic cultures are often the only practical way to maintain certain protists and to study complex ecological interactions (Jiménez‐González et al. 2025). They have additionally proven highly informative for identifying microbial partners and characterizing their functional roles within symbiotic systems (George et al. 2020; Treitli et al. 2023; Méndez‐Sánchez et al. 2024). Consequently, xenic cultures frequently represent not simply incomplete cultivation systems, but biologically meaningful experimental models. For many protists, successful cultivation may ultimately depend not on eliminating biological interactions, but on reconstructing them in experimentally tractable ways.

### 2.7 Growth, Maintenance, and Long‐Term Preservation of Protist Cultures

Protist cultures are dynamic biological systems that change over time. In the short term, nutrients within batch cultures may become depleted either by the target protist or by associated prey organisms and microbial communities. Over longer periods and repeated passages, the composition of initially mixed cultures frequently changes as co‐isolated bacteria, archaea, viruses, and eukaryotes are selectively lost, replaced, or outcompeted. Simultaneously, the target protist itself experiences strong selection under laboratory conditions, which may be accompanied by the absence of sexual reproduction, or the activation of latent viruses. Long‐term maintenance therefore represents not only a technical challenge, but also a biological one, as cultures may progressively diverge from natural populations through ecological and evolutionary change. These processes can lead to genomic or epigenetic modifications, altered physiology or behavior, including the loss of entire life history stages such as the ability to form cysts, loss of symbiotic partners or prey organisms, and eventual decline of culture viability. In practice, protist cultures sometimes collapse months or years after isolation with no immediately obvious proximate cause.

To maintain active growth and replenish depleted nutrients, cultures generally require regular transfer into fresh medium. This may involve transferring a portion of the culture into a new vessel containing fresh medium, typically at dilution ratios ranging from approximately 1:1–1:20, depending on the growth characteristics of the organism. Alternatively, concentrated nutrient solutions may be added directly to the existing culture or, for attached or benthic species, the liquid medium may simply be replaced while leaving cells adhered to the vessel surface. The frequency of transfers varies widely among taxa, ranging from daily for rapidly growing organisms such as the parabasalid *Tritrichomonas foetus* to yearly for highly resistant cyst‐forming species (ATCC 2025). When cultures are transferred into new vessels, previous cultures are often retained for days to months as backup material in case of contamination or culture collapse (Guillard and Morton 2004).

Long‐term maintenance under continuous cultivation may additionally promote laboratory adaptation and strain degeneration. Cultured protists can progressively acquire traits advantageous under artificial conditions but less representative of natural populations, including altered growth rates, morphology, metabolism, symbiotic interactions, infectivity, or life‐cycle characteristics. Preservation strategies are therefore essential not only to maintain viability but also to minimize biological drift and ensure reproducibility across experiments, laboratories, and years. Culture collections play a major role in this process by preserving reference strains together with associated metadata and standardized cultivation protocols.

Cryopreservation is among the most widely used approaches for long‐term preservation of protist cultures (McGrath et al. 1978; Caron 1993). In most cases, cells are frozen in the presence of cryoprotectants that reduce damage caused by ice crystal formation during freezing. Common cryoprotectants include dimethyl sulfoxide (DMSO), glycerol, and methanol, typically used at final concentrations between 1% and 10%. Because these compounds may themselves be cytotoxic, their effects are usually tested both before freezing and following recovery after thawing. Freezing is generally performed under controlled cooling conditions, either using specialized freezing devices or insulated containers containing liquids with high thermal inertia, such as isopropyl alcohol, to ensure gradual temperature decrease. Frozen cultures are subsequently stored in liquid nitrogen, either in the liquid or vapor phase, or in ultralow‐temperature freezers.

Recovery from cryopreservation typically involves rapid thawing until ice crystals disappear, followed by transfer into fresh medium at high dilution in order to reduce the concentration of cryoprotectants. Immediately after thawing, protist cells are often highly sensitive to environmental stress. Consequently, recovery conditions are frequently moderated using diluted medium, reduced light intensity, or temporary incubation in darkness for photosynthetic species. Cryopreservation success varies substantially among taxa, and optimization of freezing rates, cryoprotectants, and recovery conditions often remains highly species‐specific.

Beyond preservation of individual isolates, cryopreservation approaches have also been applied to natural microbial and protistan communities in order to preserve community composition and genetic diversity for downstream analyses. For example, cryopreservation protocols have been successfully applied to photosynthetic picoeukaryote assemblages collected from marine environments (Kawachi et al. 2016). Such approaches may additionally prove valuable during field sampling campaigns, where shipboard or in situ cryopreservation of environmental communities could preserve fragile or transient assemblages prior to subsequent isolation, cultivation, or molecular characterization under controlled laboratory conditions.

As an alternative to cryopreservation, some protists can be preserved in naturally resistant dormant stages such as cysts. Encystment may occur spontaneously or be experimentally induced through environmental stress, including nutrient limitation or evaporation of the culture medium. For parasitic protists such as *Giardia intestinalis* and *Entamoeba invadens* , encystment can additionally be stimulated by reproducing gut‐like environmental conditions (Sanchez et al. 1994; Einarsson et al. 2016). These dormant stages can often survive extended storage periods and may provide convenient preservation strategies for species that are difficult to cryopreserve. Effective preservation strategies are therefore essential not only for maintaining culture viability but also for ensuring long‐term reproducibility and conservation of protist diversity.

### 2.8 Why Are Some Species Still Recalcitrant to Culturing?

Despite major advances in cultivation techniques, large fractions of protist diversity remain uncultured, including some taxa that are abundant in the environment and readily detected by microscopy or environmental sequencing. Examples include rhizarians such as Acantharea and Collodaria, as well as numerous environmentally detected groups including the majority of MArine STramenopile (MAST) lineages. First, it is important to note that many protist species may remain uncultured simply because targeted cultivation efforts have never been attempted or have been extremely limited, particularly for taxa lacking obvious ecological or economic importance. In addition, many protists inhabit environments that are difficult both to sample and to reproduce experimentally. Many protists currently regarded as “unculturable” may be better viewed as “not yet successfully cultivated”, reflecting current limitations in cultivation effort, ecological understanding, and experimental methodology rather than intrinsic biological impossibility.

Where cultivation efforts have been unsuccessful, this likely reflects an incomplete reproduction of the environmental and biological conditions required for growth, survival, and completion of the life cycle (del Campo, Balagué, et al. 2013). Critical factors may include inappropriate physical or chemical parameters, unsuitable nutrient composition, the absence of essential prey or microbial partners, lack of permissive conditions for obligate life history stages and/or sexual reproduction, or failure to reproduce environmental fluctuations and periodic cues that regulate developmental transitions. In many cases, cultivation may additionally depend on poorly understood ecological interactions, including metabolites or signaling molecules produced by associated organisms. Success can also be influenced by stochastic factors such as the physiological state of isolated cells, the timing of isolation relative to the life cycle, or the accidental co‐isolation of beneficial microorganisms.

Some protists possess highly specialized ecological requirements that are difficult to replicate in laboratory conditions. For example, testate amoebae require diverse materials to construct their shells. While some species are generalists and can utilize a broad range of particles, others exhibit highly specific requirements. Certain species of the genus *Phryganella* depend on diatoms both as prey and as a source of shell material necessary to complete their life cycle (Dumack et al. 2019). Similarly, many photosymbiotic protists require conditions that simultaneously support both host and symbiont growth. This is the case for numerous acantharians, which maintain photosynthetic symbionts *in hospite* (Decelle et al. 2012). In even more complex situations, protists may depend on metabolites or signals produced by organisms with which they do not maintain direct physical associations, making these interactions difficult both to detect, and to reproduce experimentally.

Life‐cycle complexity may represent an additional barrier to long‐term cultivation. Many protists reproduce asexually under laboratory conditions, but prolonged absence of sexual or meiotic recombination may contribute to the accumulation of deleterious mutations and progressive decline in fitness. However, the environmental or physiological conditions required to trigger sexual reproduction are often unknown or difficult to reproduce experimentally. Protist life cycles may be haplontic, diplontic, or haplodiplontic (Simons and Van Beem 1987; Niklas and Kutschera 2010; Von Dassow and Montresor 2011), and many species still lack any described sexual stage (Lahr et al. 2011), further complicating efforts to establish stable long‐term cultures.

Some protists may additionally be genuinely difficult to cultivate because of extreme metabolic dependence on hosts or symbiotic partners. This is particularly true for obligate intracellular parasites, whose genomes may lack key biosynthetic pathways required for independent growth. In such cases, successful cultivation may require highly specialized host‐cell systems or co‐culture approaches that remain technically challenging.

## 3 Innovations for Protist Culturing

Recent technological and methodological advances are rapidly expanding the scope of protist cultivation. Although major progress has been achieved since the turn of the millennium, large fractions of protist diversity remain unsampled or uncultured, particularly in physically inaccessible, or environmentally extreme habitats. At the same time, many emerging cultivation approaches originally developed for bacteria, including high‐throughput isolation, microfluidics, co‐culture systems, and environmentally realistic incubation strategies, are increasingly being adapted for protist research.

Current cultured diversity remains strongly shaped by historical and technical biases toward species that are easy to collect, medically important, or readily maintained under standard laboratory conditions, often favoring fast‐growing taxa (Weissman et al. 2021). New cultivation approaches may therefore help expand protist research far beyond these historically tractable organisms. Some of the greatest remaining challenges involve protists inhabiting environments that are difficult both to sample and to reproduce experimentally, including the deep ocean, polar ecosystems, and high‐altitude habitats. We begin by discussing recent advances in deep‐sea protist cultivation.

### 3.1 Exploring Uncharted Environments: Deep Sea Sediments

Metabarcoding studies have revealed exceptional protist diversity in marine sediments, particularly in the bathyal and abyssal zones, including many previously unknown eukaryotic lineages (Cordier et al. 2022). These ecosystems therefore represent promising frontiers for future protist cultivation efforts. Of particular interest are autochthonous deep‐sea organisms, as opposed to shallow‐water or planktonic taxa that may have sedimented from the water column or exhibit broad ecological distributions (Schoenle et al. 2020).

Successful cultivation of deep‐sea protists requires not only access to these environments but also maintenance of near‐native physical and chemical conditions from sampling through incubation. Colonization substrates consisting of small PVC devices filled with nutrients have been successfully deployed for deep‐sea metazoans and may similarly facilitate enrichment of sediment‐associated protists by selectively attracting organisms migrating from surrounding sediments (Gaudron et al. 2010). In situ incubation systems and environmentally realistic enrichment approaches may therefore become increasingly important for isolating authentic deep‐sea communities.

Most deep‐sea organisms, excluding many taxa associated with hydrothermal systems, are adapted to both low temperature and high hydrostatic pressure. Rapid decompression during sampling may therefore damage cells, alter physiology, or prevent successful cultivation of obligate piezophiles. Consequently, pressure‐retaining sampling devices and incubation systems are likely to become increasingly important for successful recovery and cultivation of authentic deep‐sea taxa. For example, the PERISCOP system developed for transporting deep‐sea metazoans can also be used to retrieve colonization substrates while maintaining pressure conditions during ascent (Shillito et al. 2023). Various specialized pressure‐retaining cultivation systems are additionally available for maintaining organisms under elevated hydrostatic pressure in the laboratory (Wang et al. 2022).

To date, most deep‐sea protists have been isolated under atmospheric pressure using conventional cultivation approaches and only subsequently shown to grow more efficiently under elevated pressure conditions (Živaljić et al. 2018). This suggests that current cultivation strategies may strongly underestimate the diversity and physiology of obligately piezophilic protists. As in many other areas of protist cultivation, future advances will likely depend heavily on adapting methodologies originally developed for prokaryotes, fungi, and metazoans to the specific ecological and physiological requirements of protists (Picard and Daniel 2013).

### 3.2 High‐Throughput Culturing Methods and Microfluidics

Recent advances in microbial cultivation increasingly combine high‐throughput experimentation, microfluidics, in situ incubation, and genome‐informed prediction to expand the diversity of organisms that can be maintained in culture. Two broad and complementary strategies can be distinguished. Top‐down approaches rely on experimentally screening large numbers of environmental conditions, enrichment strategies, or cultivation systems in parallel in order to recover organisms capable of growth under at least some tested conditions. In contrast, bottom‐up approaches use genome‐, transcriptome‐, or metabolome‐informed reconstruction of physiological requirements to predict suitable cultivation conditions for specific target organisms, increasingly coupled with machine learning approaches for media optimization and interaction prediction. Both strategies have been applied successfully in bacteriology and mycology (Lewis et al. 2021; Rämä and Quandt 2021) and hold substantial promise for protist cultivation.

Top‐down cultivation approaches are particularly amenable to miniaturization and large‐scale parallelization. Isolation devices such as iChip and FiChip (Nichols et al. 2010; Marcolefas et al. 2019; Li et al. 2023) consist of microwell arrays loaded with highly diluted environmental samples such that individual wells ideally receive single cells. The devices are then sealed with semi‐porous membranes that prevent cell escape while allowing diffusion of nutrients and signaling compounds from the surrounding environment. The chips are then returned to the original habitat, including sediments, soils, or aquatic environments, allowing in situ cultivation under environmentally realistic conditions that may facilitate access to unknown growth factors or microbial interactions.

Other high‐throughput systems focus on automated cultivation and screening under tightly controlled laboratory conditions. Platforms such as Prospector (Liang et al. 2020) enable parallel cultivation in thousands of microwells under oxic or anoxic conditions with automatic fluorescent or colourimetric detection of growth. The microfluidics‐based SlipChip (Ma et al. 2014) confines cells in many parallel tiny microcompartments, which provide different but precisely controlled conditions. After an incubation period, the chip is split into two replicas, one used for species identification and the other for continued culture growth. Nanoporous microscale microbial incubators (Ge et al. 2016) similarly allow stochastic seeding of individual cells into chemically connected but physically isolated chambers, facilitating systematic exploration of co‐culture interactions and microbial dependencies. More broadly, automated high‐throughput culture platforms developed for stem cell biology, tissue engineering, and drug screening increasingly permit systematic optimization of environmental parameters across thousands of parallel growth conditions (Ryoo et al. 2024). Depending on the platform, cultivation may occur in microplates, microarrays containing distinct chemical cues, or highly integrated microfluidic devices. The major challenge is now to adapt these approaches to the exceptional biological diversity of protists. Unlike many bacterial systems, successful protist cultivation frequently requires maintaining complex ecological interactions, including predator–prey dynamics, symbioses, and chemically mediated microbial associations. Consequently, cultivation often involves not merely maintaining a single organism, but recreating a functional ecological system. This substantially complicates miniaturization and physical isolation strategies. Protists additionally exhibit extraordinary variation in cell size, morphology, motility, and life cycles, ranging from picoeukaryotes to giant multinucleate cells, making the design of universal cultivation platforms particularly difficult.

Nevertheless, initial progress has already been made. Microfluidic devices have been developed to trap and observe cells of *Chlamydomonas*, *Giardia*, and trypanosomes (Lu et al. 2017; Széles et al. 2022; De Niz et al. 2023), evolutionarily important protists such as *Picomonas judraskeda*, the first identified picozoan (Seenivasan et al. 2013), have been isolated by FACS, a technique which promises to contribute to high‐throughput isolation approaches (del Campo et al. 2024), while other systems have been used to investigate protist ecology in soil microenvironments (Mafla‐Endara et al. 2021). Although these platforms have not yet been widely adapted for long‐term protist cultivation, they illustrate the substantial potential of microfluidic and high‐throughput technologies for future protistology. As single‐cell omics, machine learning, automated imaging, and environmental simulation technologies continue to advance, high‐throughput cultivation approaches may fundamentally transform the scale and diversity of protists accessible for laboratory study.

### 3.3 Genome‐ and Transcriptome‐Informed Approaches

The identification of appropriate media for culturing protists is still largely based on extensive trial and error and requires significant expertise. However, the increasing availability of genomic, transcriptomic, metabolomic, and environmental sequencing data is beginning to enable more predictive approaches to cultivation design. In principle, reconstruction of metabolic pathways from omics datasets can help identify nutritional requirements, missing biosynthetic capacities, environmental tolerances, and potential ecological dependencies of target organisms. Nevertheless, translating such predictions into successful cultivation strategies remains highly challenging.

Genome‐informed approaches have already been applied successfully in bacteriology and mycology. Genomic information has been successfully used to infer bacterial preferences across oxygen gradients (Jabłońska and Tawfik 2019) and temperature regimes (Sauer and Wang 2019). Detailed metabolism reconstruction has additionally enabled the identification of missing pathways and has resulted in the prediction of appropriate media composition for previously uncultured oligotrophic bacteria (Carini et al. 2013). In another example, genomic reconstruction, microarray analysis, and metabolite profiling of the pathogenic bacterium *Coxiella burnetii* allowed the development of a complex nutrient medium which permitted axenic (host‐free) culturing (Omsland et al. 2009).

Accumulated genomic datasets, growth curves, and media preference information also provide increasingly valuable training data for machine learning approaches aimed at predicting cultivation conditions. For example, machine learning models trained on bacterial genomic information have successfully predicted pH preferences even in cases where no strong phylogenetic signal was observed (Ramoneda et al. 2023). Instead, specific genes and metabolic traits associated with environmental adaptation could be identified across unrelated lineages. Similar approaches may eventually help predict optimal nutrient composition, oxygen tolerance, salinity, temperature, or co‐culture requirements directly from genomic data, although substantial technical and conceptual challenges remain (Hashizume and Ying 2024).

Application of these approaches to protists presents additional complexities. Protist genomes are often large, highly compartmentalized, and incompletely characterized, and many species exhibit extensive metabolic flexibility or depend on prey organisms, symbionts, or microbial communities for growth. Consequently, predicting cultivation conditions for protists may require reconstruction not only of individual metabolism but also of ecological interactions and community context. Metagenome‐assembled genomes, single‐cell genomes, and single‐cell transcriptomes are particularly important for currently uncultured protists precisely because they provide access to genomic information in the absence of established cultures; although this information is incomplete due to the partial nature of single‐cell or metagenome assemblies, it can provide an initial starting point to understand metabolic or other growth requirements.

At present, a major circular dependency limits progress. Genomic information from uncultured protists would greatly facilitate prediction of appropriate cultivation conditions, yet high‐quality reference genomes are still most readily obtained from cultured species. This limitation complicates assembly and interpretation of protistan metagenome‐assembled genomes from environmentally detected taxa. Continued advances in sequencing technologies, single‐cell approaches, bioinformatics, and high‐throughput cultivation platforms will likely help progressively overcome this barrier.

Equally important may be the reconstruction of the chemical and microbial environment surrounding the target organism. Successful cultivation may depend not only on supplying appropriate nutrients, but also on reproducing metabolites, signaling compounds, or ecological interactions generated by surrounding microbial communities. Integrating environmental metabolomics, chemical profiling, and microbial community metabolic reconstruction may therefore become essential for rational media design and prediction of cultivation conditions (Jiménez‐González et al. 2025). For many protists, successful cultivation may ultimately require predicting not only the nutritional requirements of individual species but also the ecological and metabolic interactions occurring within their surrounding microbial communities.

### 3.4 More Universal Media

The extraordinary diversity of protist nutritional strategies has resulted in the development of a large number of cultivation media, many optimized for relatively narrow phylogenetic, or ecological groups. In most cases, cultivation media are designed to reproduce the natural growth environment of the target organism as closely as possible. A relatively small number of broadly permissive media have nevertheless been used successfully to cultivate diverse phototrophic and heterotrophic protists (Supplementary Table 1). However, many uncultured taxa likely require more specialized or currently unknown nutritional conditions.

Developing suitable media for these organisms generally involves extensive empirical testing of different nutrient compositions, trace elements, vitamins, organic substrates, and environmental conditions. This has led to the proliferation of lineage‐ or habitat‐specific media formulations (Supplementary Table 2), some of whose formulations have not been revisited for decades. Consequently, an important challenge for future protistology is the development of more broadly applicable screening media capable of supporting growth of phylogenetically and ecologically diverse taxa during initial isolation and enrichment steps. Such media would not necessarily replace optimized formulations for long‐term cultivation or physiological experiments, but could substantially facilitate high‐throughput screening and recovery of previously uncultured diversity.

One possible strategy is to combine components from existing successful media into chemically defined formulations designed to satisfy the requirements of a wide range of protists simultaneously. One recent attempt to develop such a broadly permissive medium (RS medium) successfully supported the growth of phototrophic and heterotrophic protists representing 10 major eukaryotic lineages and enabled cultivation of new species of telonemids, kinetoplastids, choanoflagellates, amoebozoans, and goniomonads (Mostazo‐Zapata et al. 2025; Packer et al. 2025; Zavadska et al. 2025, 2026; Gàlvez‐Morante, Berney, and Richter 2026; Gàlvez‐Morante, Rutaganira, et al. 2026). More generally, modular media designs consisting of standardized base formulations supplemented with variable additives may provide a flexible compromise between universality and taxon‐specific optimization.

A complementary and more data‐driven strategy is to systematically map the growth of established cultures across large numbers of known media in order to identify media with the broadest applicability or predict novel organism‐medium pairings. Similar approaches have already been explored in bacteriology, where databases integrating known strains, media compositions, and cultivation outcomes have been used to predict successful cultivation conditions (Oberhardt et al. 2015). One major component of this effort involved standardizing and codifying media formulations in searchable formats. The resulting database included more than 18,000 bacterial species, over 3000 media formulations, and more than 20,000 organism‐medium associations, enabling identification of both broadly permissive and highly specialized media across the bacterial tree of life. Experimental validation of predicted organism‐medium pairings demonstrated high success rates, highlighting the potential of “growth medium transitivity,” whereby successful cultivation conditions for related organisms can help predict suitable media for previously uncultured taxa.

A similar effort could prove highly valuable for protists, for which no comprehensive searchable database of organism‐medium pairings currently exists. However, the prediction of cultivation requirements based on phylogenetic relationships may be substantially more challenging in eukaryotes because of the ecological complexity, and the prevalence of trophic flexibility, symbiosis, and prey dependence among protists.

## 4 Conclusion

Although protists were among the first microorganisms ever observed and cultivated (Leeuwenhoek 1677) (see Box 1), protist cultivation has historically received far less attention than bacterial or fungal cultivation despite the central evolutionary and ecological importance of these organisms. Nevertheless, substantial recent progress has transformed the field, with advances in environmental sampling, cultivation technologies, genomics, microfluidics, high‐throughput screening, and computational biology increasingly expanding the diversity of protists accessible to laboratory study.

In this review, we summarize current knowledge and methodologies for the cultivation of phylogenetically and trophically diverse protists, spanning isolation, enrichment, identification, routine maintenance, and long‐term preservation. Across these approaches, several common themes emerge: successful cultivation frequently depends on reproducing ecological interactions and life‐cycle transitions, many currently uncultured taxa may simply not yet have been tested under appropriate conditions, and future progress will increasingly rely on integrating ecological, physiological, and genomic information into predictive cultivation strategies. We additionally highlight both common cultivation principles and important biological exceptions that reflect the extraordinary diversity of protist lifestyles and environmental adaptations.

The convergence of high‐throughput cultivation, environmental sequencing, machine learning, metabolomics, and microengineering approaches is beginning to shift protist cultivation from a largely empirical discipline toward a predictive and scalable science. As these approaches continue to develop, they may profoundly expand the diversity of protists available for experimental investigation, with major implications for understanding eukaryotic evolution, microbial ecology, symbiosis, biogeochemical cycling, and the functioning of the biosphere.

## Funding

This work was supported by the Czech Science Foundation grants 23‐06479X (to J.L.), 23‐07277S (to V.H.) and the European Commission Horizon Europe project BIOservicES (grant agreement 101112374). This project has received funding from the European Research Council (ERC) under the European Union's Horizon 2020 research and innovation programme (grant agreement No. 949745), from the Departament de Recerca i Universitats de la Generalitat de Catalunya (exp. 2021 SGR 00751), and from grant PID2023‐152955NA‐I00 funded by MICIU/AEI/10.13039/501100011033 and by ERDF/EU (to D.J.R.). This work was supported by the National Science Centre, Poland (Opus grant 2020/37/B/NZ8/01456 to A.K. and WEAVE‐UNISONO grant 2021/03/Y/NZ8/00076 to K.P.). E.L. was partially funded by a grant from the Spanish Government from the Program “Generación de Conocimiento” PID2021‐128499NB‐I00 10.13039/501100011033/, (MCIU/AEI/FEDER, UE). C.R.‐M. was partly funded by the UK Natural Environment research Centre (project NE/Y006321/1). I.P. was supported by the French National Research Agency through the PHENOMAP project (ANR‐20‐CE02‐0025) and by the European Union's Horizon Europe research and innovation programme under Grant Agreement No. 101082304 (BlueRemediomics).

## Conflicts of Interest

The authors declare no conflicts of interest.

## Supporting information


**Supplementary Table 1.** Common media recipes for growing heterotrophs and phototrophs.
**Supplementary Table 2**. Links to organization websites hosting collections of media recipes.
**Supplementary Table 3**. List of worldwide culture collections containing protists.

## Data Availability

Supplementary tables and the tree presented in Figure 1a are available on GitHub: https://github.com/beaplab/protist_culturing_guide.
